# Editorial: Cancer systems biology

**DOI:** 10.3389/fgene.2022.1083902

**Published:** 2022-11-18

**Authors:** Jesús Espinal-Enríquez, Claudia Rangel-Escareño, Frank Emmert-Streib

**Affiliations:** ^1^ Computational Genomics Division, National Institute of Genomic Medicine, Mexico City, Mexico; ^2^ School on Engineering and Sciences, Tecnologico de Monterrey Epigmenio González, Santiago de Querétaro, Mexico; ^3^ Predictive Society and Data Analytics Lab, Tampere University, Tampere, Finalnd

**Keywords:** cancer systems biology, cancer Metabolism, biomarkers in cancer, cancer immunobiology, non-coding regulation in cancer

## Introduction

Cancer has been a paradigmatic example of a complex disease. It is the second leading cause of death worldwide. Knowledge acquired on the mechanisms and potential causes underlying the origins and progression of cancer from single-scale studies have led to the development of personalized treatments, which in turn have decreased mortality rates in several types of cancer. However, because it is not a single and isolated disease, but rather a heterogeneous set of multi-scale alterations, current research requires computational and systems biology approaches that deal with complex systems on gene regulatory networks. Multi-omics is one of these approaches. Aimed at developing multi-scale mathematical modeling, it allows us to integrate the dynamics of biological perturbations for different types of cancer. Moreover, if tissue/organ-specific path is studied we are able to create mechanistic models for a particular type of the disease. That, combined with current low-cost high throughput technologies have made possible massive cohorts of -omics and clinical data to be publicly available. This immeasurable amount of information promotes harnessing data science approaches for the analysis, integration, and mining of multi-omics data. Large and multidisciplinary groups can now focus on creating models and provide theoretical frameworks that describe cancer in a variety of ways. Besides cancer cell transformation or cancer evolution, currently single cell genomics and spatial single cell transcriptomics is moving forward with deeper understanding of the disease. Also, cancer driver mutations and mutational processes, development of target-specific treatments have been leading to a prediction of drug responses. Notwithstanding, several challenges that remain to be undertaken such as mechanisms of metastasis, resistance to treatment, intra-tumoral heterogeneity, molecular, cellular, and metabolic changes during progression stages, epigenetic modifications, to mention a few. This cross-disciplinary interaction at these levels of description have contributed to develop more efficient models that improve the predictive capacity and ultimately help clinicians and medical scientists in the treatment and therapies.

The aim of this Research Topic is to discuss and explore state-of-the-art research on cancer systems biology. Throughout this Research Topic of articles, several aspects of cancer systems biology are covered. On the one hand, the cancer-specific point of view, which tries to identify molecules, pathways, mechanisms that generate or alter a concrete type of cancer, in one tissue. On the other hand, the multi-cancer point of view, which tries to observe generalities, common aspects, or the widely mentioned hallmarks of cancer. Also, several bioinformatics tools were implemented to dissect and discriminate between groups and to observe specific patterns. Among these computational tools we can find language-specific or web-based bioinformatic packages such as TIMER, GEPIA2, LinkedOmics, GSCALite, GSEA, CIBERSORT, ESTIMATE, WGCNA ARACNe, or Infotheo, among others. Tissue-specific approach was widely used in mainly four applications: cancer metabolism, biomarkers and prognosis, immune system-related research and, non-coding regulation. A highlight of this Research Topic is that the majority of manuscripts focus on establishing a better prognosis or a more accurate classification of cancer groups. We summarize the 15 articles accepted for publication in the systems biology Research Topic.

## Cancer metabolism

Zhu et al. studied the relationships between glycolysis, tumor microenvironment, and therapeutic response in esophageal adenocarcinoma (EAC). By classifying into low and high risk groups and based on a glycolysis-related genes signature, these authors established a correlation between glycolytic and ATP/ADP metabolic pathways and a poor overall survival. Wang et al. systematically analyzed the expression patterns of lysyl oxidase family genes in gastric cancer. They found that LOX and LOX2 may have a role in tumor prognosis and therefore, in gastric cancer therapy. Sun et al. investigated the association between COX-2 –1195G/A single nucleotide polymorphism and lung cancer susceptibility in a Japanese population. By evaluating the genotype distribution of COX-2 –1195G/A with a PCR-restriction fragment length polymorphism assay for 330 lung cancer patients and 162 healthy controls, they show that COX-2 –1195G/A does not have a relationship with the risk of developing lung cancer. However, homozygous COX-2–1195A genotype confers an increased risk for lung squamous cell carcinoma in Japanese individuals and could be used as a predictive factor for early detection of lung squamous cell carcinoma.

## Biomarkers and prognosis

Regarding biomarkers and prognosis, Chen et al. evaluated the correlation between NCKAP1 expression and clinical features of clear cell renal cell carcinoma. These authors found that overexpression of NCKAP1 in ACHN cells reduced proliferation, invasion and migration capacity *in vitro* and inhibited tumor growth *in vivo*. Hu et al. explored the molecular mechanism of LYPD3 in the regulation during transformation and throughout the development of acute myeloid leukemia providing a research basis for the screening of markers related to the treatment and prognosis. They specifically suggest, by means of a dataset analysis and gene knockdown mediated by small interfering RNA (siRNA), that LYPD3 participates in the development of AML through the p53 signaling pathway or/and PI3K/AKT signaling pathway. Zhou et al. assessed the diagnostic and prognostic significance of ATP binding cassette subfamily C (ABCC) genes in hepatocellular carcinoma (HCC). ABCC1,4,5 were found to be positively associated with infiltration of multiple immune cells, while ABCC6 was found to be the opposite. In conclusion, they suggest that ABCC1, ABCC4, ABCC5, and ABCC6 might be prognostic biomarkers in HCC. Zhang et al. explored the expression and carcinogenic effect of keratin 17 (KRT17) in human tumors. They show that KRT17 was highly expressed in most tumors (such as esophageal cancer, lung cancer, cervical cancer, *etc.*), and the high expression level correlated with tumor stage and prognosis. Zheng et al. evaluated the relationship between EFNA3 and gastric cancer (GC) prognosis and tumor-infiltrating lymphocytes. The authors suggest based on bioinformatics analyses, that EFNA3 participates in changes in GC immune checkpoint markers in a co-linear manner. Turns out that EFNA3 expression in HGC-27, AGS, MKN45, and NCI-N87 cell lines was higher than that in GES-1 and observed that patients with high expression of EFNA3 had a worse prognosis.

## Cancer and immune system

Among the immune-system related works, Zhang S. et al. constructed a prognostic model for the response to immunotherapy in thyroid carcinoma. The authors show that patients with high tumor mutational burden and low PD-L1 expression levels might respond poorly to immunotherapy. Yang S. et al. identified three different immune cell infiltration signatures. The one with the highest risk was characterized by an enhanced activation of the immune system as well as a significantly high tumor mutational burden. Xie et al. evaluated the thymidine kinase 1 (TK1) role in prostate cancer databases with results validated for *in vitro* and *in vivo* models. They showed that TK1 is a prognostic predictor correlated with poor outcomes of PCa patients. Moreover, TK1 inactivation can significantly restrain tumor growth. Zhang Y. et al. constructed a prognostic model based on pyroptosis-related genes to provide new insights into the prognosis of osteosarcoma patients. Based on a pyroptosis-related signature score, they were able to differentiate patients with high and low risk of metastasis.

## Non-coding regulation

In terms of gene expression correlating with other omics, Hernández-Gómez et al. evaluated the role that CNVs may play in shaping gene co-expression patterns in luminal B breast cancer. The authors construct a conditional mutual-information-based network for gene expression and copy number alterations. They report that, for luminal B breast tumors, the co-expression program is not necessarily determined by its CNV structure. Additionally, by analyzing the network topology, they suggested that MAF1 and POLR3D may constitute an axis of regulation of gene transcription, in particular for non-coding RNA species. Methylation is a widely known mechanism involved in cancer development. Shi et al. analyzed the m6A RNA methylation in several types of cancer. The results revealed that the m6A regulators exhibited widespread dysregulation, genetic alteration, and the modulation of oncogenic pathways across cancer types. In addition, most of the m6A regulators were relevant for prognosis in many cancer types. It has been recently shown that long non-coding RNAs play a crucial role in the development of several cancer types. Yang X. et al. developed a pyroptosis-related lncRNA signature to evaluate and predict overall survival in breast cancer. The risk model comprised 10 pyroptosis-related lncRNAs, and was identified as an independent predictor for overall survival (OS). The low-risk group had a higher expression of immune checkpoint markers and exhibited higher fractions of activated immune cells, while the high-risk group had a higher percentage of TMB. A validation on a separate cohort of breast cancer samples found that RP11-459E5.1 was significantly upregulated, while RP11-1070N10.3 and RP11-817J15.3 were downregulated and significantly associated with worse OS.

Altogether, this Research Topic of 15 articles provides a broad overview of the current status of cancer systems biology, showing promising advances of cancer research through the application of computational approaches.

